# Distinguishing between translational science and translational research in CTSA pilot studies: A collaborative project across 12 CTSA hubs

**DOI:** 10.1017/cts.2023.700

**Published:** 2023-12-18

**Authors:** Margaret Schneider, Amanda Woodworth, Marissa Ericson, Lindsie Boerger, Scott Denne, Pam Dillon, Paul Duguid, Eman Ghanem, Joe Hunt, Jennifer S. Li, Renee McCoy, Nadia Prokofieva, Vonda Rodriguez, Crystal Sparks, Jeffrey Zaleski, Henry Xiang

**Affiliations:** 1 The Institute for Clinical and Translational Science, University of California, Irvine, CA, USA; 2 The Institute of Translational Health Sciences, University of Washington, Seattle, WA, USA; 3 The Indiana Clinical and Translational Sciences Institute, Indiana University, Indianapolis, IN, USA; 4 The Wright Center for Clinical and Translational Research, Virginia Commonwealth University, Richmond, VA, USA; 5 The Translational Research Institute, University of Arkansas Medical Sciences, Little Rock, AR, USA; 6 Duke Clinical & Translational Science Institute, Duke University, Durham, NC, USA; 7 Clinical & Translational Science Institute of Southeast Wisconsin, Medical College of Wisconsin, Milwaukee, WI, USA; 8 Tufts Clinical and Translational Science Institute, Tufts University, Boston, MA, USA; 9 Center for Clinical and Translational Science, The Ohio State University, Columbus, OH, USA

**Keywords:** Translational science, translational research, principles, generalizability, efficiency

## Abstract

**Introduction:**

The institutions (i.e., hubs) making up the National Institutes of Health (NIH)-funded network of Clinical and Translational Science Awards (CTSAs) share a mission to turn observations into interventions to improve public health. Recently, the focus of the CTSAs has turned increasingly from translational research (TR) to translational science (TS). The current NIH Funding Opportunity Announcement (PAR-21-293) for CTSAs stipulates that pilot studies funded through the CTSAs must be “focused on understanding a scientific or operational principle underlying a step of the translational process with the goal of developing generalizable solutions to accelerate translational research.” This new directive places Pilot Program administrators in the position of arbiters with the task of distinguishing between TR and TS projects. The purpose of this study was to explore the utility of a set of TS principles set forth by NCATS for distinguishing between TR and TS.

**Methods:**

Twelve CTSA hubs collaborated to generate a list of Translational Science Principles questions. Twenty-nine Pilot Program administrators used these questions to evaluate 26 CTSA-funded pilot studies.

**Results:**

Factor analysis yielded three factors: Generalizability/Efficiency, Disruptive Innovation, and Team Science. The Generalizability/Efficiency factor explained the largest amount of variance in the questions and was significantly able to distinguish between projects that were verified as TS or TR (*t* = 6.92, *p* < .001) by an expert panel.

**Conclusions:**

The seven questions in this factor may be useful for informing deliberations regarding whether a study addresses a question that aligns with NCATS’ vision of TS.

## Introduction

In the last decade of the 20^th^ century, translational research started to gain momentum in biology and medicine [1]. While the scientific community, policymakers, and the general public value and support basic research whose primary goal is to build the scientific basis for the development of novel therapies, there have been longstanding concerns that much basic research has little immediate impact on clinical practice and public health interventions [2–4]. A previous study reported the estimated time lag between journal publication of a significant basic science discovery to use in practice was between 17 and 23 years [5]. This long lag time was corroborated by a study that examined more than 15 million Medline articles published between 1980 and 2013 [6]. Recognizing this challenge and in response to the push for more timely benefits to the clinical practice, patient outcomes, and population health, countries around the world have initiated major programs aiming to speed up the movement of promising scientific discoveries to clinical practice or public health interventions [2,3,7]. Since 2005, the National Institutes of Health (NIH) in the United States, the Medical Research Council of the United Kingdom, the Korean Ministry of Health and Welfare in South Korea, and the Chinese Academy of Medical Sciences in China, to name a few, have established major funding streams to support translational research [2].

The National Center for Advancing Translational Sciences (NCATS) was established in 2011 by the NIH to “*transform the translational process so that new treatments and cures for diseases could be delivered to patients faster* [3,4,7,8].” One major funding initiative by NCATS to support this mission is the network of academic institutions across the U.S. called Clinical and Translational Science Award (CTSA) hubs. In fiscal year 2022, there were 63 CTSA hubs and each CTSA-funded hub provided services to its institution(s) including shared research infrastructure, collaboration tools, training and educational opportunities, administrative support (e.g., streamlining IRB approvals or data safety monitoring boards), and pilot research funding programs [9]. A key component of all CTSA hubs, the CTSA Pilot Programs projects “*are intended to: (1) explore possible innovative new leads or new directions for established investigators; (2) stimulate investigators from other areas to lend their expertise in research in [clinical translational science]; and (3) provide initial support to establish proof of concept* [10].”

Originally, the CTSA Pilot Programs focused on translational research, which was defined by NCATS as the process of turning observations in the laboratory, clinic, and community into clinical practice and interventions that improve individual and public health [1,3]. According to this holistic concept, translational research is defined as the effort to traverse specific steps of the translational process for a particular target or disease. Over more than a decade, the terms translational *research* and translational *science* were used interchangeably [2,4,6,11–13]. Recently, an effort to distinguish translational research from translational science has prompted a related but different definition of translational science and stimulated the evolution of a distinct discipline [14]. NCATS currently features the following definition of translational science on its website: Translational science is the field that generates innovations that overcome longstanding challenges along the translational research pipeline. These include scientific, operational, financial, and administrative innovations that transform the way that research is done, making it faster, more efficient, and more impactful [15].

There are many misconceptions about the definitions of translational research and translational science [16,17]. Using the terms interchangeably or discussing translational science to describe translational research projects adds confusion to the field. To effectively advance the field of translational science, there is a need for clear strategies for distinguishing between these two distinct but related disciplines. Moreover, the current CTSA Funding Opportunity Announcement (FOA) [10] stipulates that pilot studies funded through the CTSAs must be focused on translational science. This new directive places CTSA Pilot Program administrators in the position of arbiters with the task of distinguishing between translational science and translational research. The purpose of this study was to leverage the collective knowledge and experience of the CTSA External Reviewer Exchange Consortium (CEREC) [18] to explore whether a set of TS principles set forth by NCATS might be useful in distinguishing TS from TR; work that will be useful to the national CTSA network to meet the NCATS mandate.

## Methods

### Procedures

Twelve CTSA hubs participated in this study (see hub descriptions in Supplementary Table 1). Pilot Program administrators (Program Directors and Program Managers) from 10 hubs submitted up to three research proposals for pilot studies that had previously been funded at their CTSA hubs. Data collection occurred prior to any of the participating hubs being funded under the new FOA. To ensure that projects of both types were represented, instructions to submitting administrators provided the NCATS definitions of translational science (TS) and translational research (TR) and requested that up to two TS projects and up to one TR project be submitted. Submissions included the abstract and research plan (limited to five pages) and were collected using Research Electronic Data Capture (REDCap®) [19,20].

A set of questions was developed to reflect TS principles as delineated by NCATS (see details in Measures) [15]. Using these Translational Science Principles questions, pilot study projects submitted by participating hub administrators were evaluated by 29 individuals (coders) administratively affiliated with the CTSA Pilot Programs at the 12 participating hubs. This task was accomplished using a second REDCap® survey that provided coders with each pilot study proposal and asked them to agree or disagree that the proposal met the objectives of each question. A CEREC coordinator assigned projects to coders in a manner that ensured no project was scored by the administrator who had submitted it, and each project was scored by at least three independent coders.

To establish whether each project met the spirit of the NCATS definition of TS, projects were subsequently evaluated by a subset of the authors identified as topic experts (see Data Analysis for details). Categorization of projects as TS or TR was determined by expert consensus, which was established using another REDCap® survey.

### Measures

#### Data source

A checklist was provided to project submitters to characterize the pilot study data source. Multiple categories could be selected, including (1) Basic Science Lab (includes research on cells, blood, and other biological products); (2) Animal Study; (3) Human Subjects Study; (4) De-identified data from human subjects (e.g., Electronic Health Record (EHR) data); and 5) Other.

#### Disciplines

A checklist was provided to project submitters to characterize the approaches or disciplines represented in the pilot study projects, including (1) Community-based participatory research; (2) Dissemination and Implementation; (3) Informatics; (4) Regulatory Processes; (5) Drug or Device Development; (6) Research Design/Statistics/Research Methods; (7) Team Science; (8) Recruitment/Retention; and (9) Other.

#### Questions relating to principles of translational science

A set of questions was constructed based on the 20 principles of TS posted on the NCATS website in September 2022 (see Table 1; full descriptions provided in Supplementary Materials). The set of questions was developed in three iterative steps. In step one, a pool of 44 items was created by the first author, comprised of items worded to preserve the complexity of the TS principles as stated on the NCATS website, as well as items that were modified to offer more streamlined versions consistent with best practices of survey design (e.g., avoiding double-barreled questions). These 44 items were circulated to the coauthors for feedback. In step two, items were removed that were redundant, unclear, or did not map onto the NCATS TS principles. Two items also were removed that referred to establishing research funding opportunities, as CTSA-funded pilot awards are not intended to set up research funding opportunities. In step three, the shorter set of 24 questions was again circulated to the coauthors, which resulted in a modification of the response options from a five-point scale (strongly disagree to strongly agree) in favor of a dichotomous scale (“agree” or “disagree”) with the option for “insufficient information to determine.” Table 1 lists the 24 questions, along with the corresponding TS principles.

**Table 1.** Translational science principles questions, corresponding translational science principles, and percent of responses indicating not enough information within the proposal to determine (N/A)

	Survey item	% N/A	TS Principle^1^
1	If successful, this project will yield information that will accelerate translational research.	6	E
2	The knowledge gained from this project will be generalizable to a variety of diseases.	10	B
3	This project addresses a common roadblock or bottleneck in translational research.	10	B
4	If successful, this project will improve translational research by making it more efficient or effective.	10	E
5	This project addresses an unmet scientific, patient or population health need.	3	A
6	This project uses a multi-disciplinary approach.	17	D
7	This project is innovative in terms of the scientific approach, methods, or processes.	10	C
8	This project is ambitious.	16	G
9	This project develops a novel method or technology.	3	C
10	This project will be impactful.	14	G
11	This project is likely to be transformative.*	25	G
12	This project contains paradigm-challenging ideas.	18	C
13	This project will develop and implement innovations in scientific approaches, methods and/or technologies to accelerate the pace of translational research.	11	E
14	This project approaches research challenges and development of solutions by seeking commonalities across research on a range of diseases and conditions.	6	B
15	This project will develop and/or implement innovations in teamwork, partnerships, and operations that will enhance the quality and impact of the research.*	23	C
16	This project engages all relevant expertise across disciplines, fields, and/or professions to produce research that advances translation.	19	D
17	This project integrates concepts, theories, methods, technologies, and/or approaches from the range of disciplines, fields, and professions that can contribute to advancing the research goals.	19	D
18	This project leverages knowledge integration to develop more holistic findings that are therefore more relevant to real-world applications.	18	D
19	This project will implement evidence-based practices to enhance the speed at which teams develop shared goals and improve team communication and coordination of work tasks.*	28	E
20	This project will implement milestone-based decision making to enable rapid agreement on go/no-go decisions, to enable resources to be used most efficiently.*	32	E
21	This project will reward efficiency, enable rapid failures and encourage redirection of resources to subsequent attempts.*	31	E
22	This project will implement evidence-informed practices for collaborations, engagement and partnership.*	27	F
23	This project will incentivize collaboration through recognition and reward systems that value team science, cross-disciplinary collaboration, patient and community engagement, and cross-agency partnerships.*	35	F
24	This project encourages transformative ideas and risk taking toward achieving the overall goal of improving the translational process.	17	E

* Excluded from factor analysis; more than 20% of responses indicated “not enough information to determine.”

1 Key to Translational Science Principles

^a^Prioritize initiatives that address unmet needs (Focus on Unmet Needs).

^b^Produce cross-cutting solutions for common and persistent challenges (Generalizable solutions).

^c^Emphasize creativity and innovation (Creativity and Innovation).

^d^Leverage cross-disciplinary team science (Cross-disciplinary Team Science).

^e^Enhance the efficiency and speed of translational research (Efficiency and Speed).

^f^Utilize boundary-crossing partnerships (boundary-crossing partnerships).

^g^Use bold and rigorous research approaches (bold and rigorous).

### Data Analysis

Prior to analysis, scores of “insufficient information to determine” on the Translational Science Principles questions were coded as missing, and seven items for which 20% or more of responses were missing were excluded from the analysis (identified with an asterisk in Table 1). A Principal Component Analysis was conducted on the remaining 17 questions using SPSS Statistics version 28 (IBM Corp., Armonk, NY). Due to the exploratory nature of this research, several model-fitting techniques were tested, including both orthogonal and oblique rotations. While an orthogonal rotation (i.e., Varimax) minimizes the number of variables with high loadings and simplifies the solution, an oblique rotation (i.e., Promax) allows components to be intercorrelated [21]. Our guiding hypothesis was that we would be able to identify at least one factor that would distinguish TS from TR and would be uncorrelated with any additional factors. As a significant dearth of similar validation studies exists in the literature – and thus no factor analytic studies with which to compare – we examined both Promax and Varimax rotations. Several criteria were used to determine the number of factors and combination of items in each factor, including a scree plot of Eigenvalues [22], item loadings [23], and Kaiser criterion [24]. The criterion cutoff was set at ± 0.35 for the item loadings. Based on this criterion, each item loaded most highly on one of three distinct factors. Cronbach’s alpha values were computed for the three factors and items were removed if they weakened the reliability of the factor.

To ascertain whether the factors could be useful for discriminating between TS and TR, expert consensus was used to label projects as TS or TR. Experts were members of the author team who met the following criteria: (1) affiliated with a hub that had submitted a CTSA application to NCATS under the most recent FOA; (2) reported having read the pilot study section of the most recent FOA; and (3) reported having discussed TS with colleagues at their hub “a fair amount” or “quite a bit.” Expert consensus was defined as at least seven out of eight experts assigning the same label (TS or TR) to a project (i.e., minimum of 87% agreement). A comparison of mean factor scores between the TS and TR projects for which expert consensus had been achieved was carried out using *t*-tests, and item-level comparisons in percent agreement were conducted using Chi-Square analyses.

## Results

### Description of Research Projects

A total of 26 research projects were submitted (see Table 2 for characteristics).

**Table 2.** Characteristics of research projects (*N* = 26)

Data source		No. (%)
	Basic science lab	4 (15)
	Animal studies	5 (19)
	Human subjects	16 (62)
	De-identified data from human subjects	1 (4)
	Other	3 (12)
**Approaches/Disciplines**		
	Community-based participatory research	6 (23)
	Dissemination and implementation	2 (8)
	Informatics	1 (4)
	Regulatory processes	1 (4)
	Drug or device development	8 (31)
	Research design/Statistics/Research methods	7 (27)
	Team science	1 (4)
	Recruitment/Retention	4 (15)
	Other	6 (23)

Multiple items could be selected. Percentages do not add up to 100%.

## Description of Coders

Twenty-nine coders participated in the study. The coders can be described as follows:

- 70% were affiliated with a CTSA hub that had submitted a grant application in response to the new FOA.
- 93% had read the section of the FOA describing the requirements for the pilot study program.
- Most had discussed TS with colleagues at their CTSA hub “A fair amount” (51%) or “Quite a bit” (29%).

### Factor Analysis

Factor analysis yielded three factors explaining 65% of the variance (see Table 3). The seven items loading on Factor One (“Generalizability/Efficiency”), which accounted for 44% of the variance, mapped onto two of the TS principles identified by NCATS: *Generalizable Solutions* and *Efficiency and Speed*. Factor Two (“Disruptive Innovation”) contained five items explaining an additional 12% of the variance and mapped onto the TS principles of *Creativity and Innovation, Bold and Rigorous*, and *Focus on Unmet Needs*. Factor Three (“Team Science”) was comprised of four items, explaining an additional 9% of variance, that mapped onto a single TS principle of *Cross-disciplinary Team Science*.

**Table 3.** Translational science principles survey items, factor loadings, and Cronbach’s alpha values

	Factor loadings			
	1 *α* =0.95	2 *α* = 0.80	3 *α* = 0.77	TS Principle
*Generalizability/Efficiency*				
Q3. This project addresses a common roadblock or bottleneck in translational research	**0.845**			Generalizability
Q2. The knowledge gained from this project will be generalizable to a variety of diseases.	**0.838**			Generalizability
Q14. This project approaches research challenges and development of solutions by seeking commonalities across research on a range of diseases and conditions.	**0.668**			Generalizability
Q4. If successful, this project will improve translational research by making it more efficient or effective.	**0.833**			Efficiency
Q13. This project will develop and implement innovations in scientific approaches, methods and/or technologies to accelerate the pace of translational research.	**0.832**			Efficiency
Q25. This project encourages transformative ideas and risk taking toward achieving the overall goal of improving the translational process.	**0.784**			Efficiency
Q1. If successful, this project will yield information that will accelerate translational research.	**0.627**			Efficiency
*Disruptive Innovation*				
Q10. This project will be impactful.		**0.751**		Bold
Q8. This project is ambitious.		**0.741**		Bold
Q7. This project is innovative in terms of the scientific approach, methods, or processes.		**0.773**		Innovation
Q9. This project develops a novel method or technology.		**0.512**		Innovation
Q5. This project addresses an unmet scientific, patient or population health need.		**0.607**		Unmet Need
*Team Science*				
Q17. This project integrates concepts, theories, methods, technologies, and/or approaches from the range of disciplines, fields, and professions that can contribute to advancing the research goals.			**0.876**	Cross-Disciplinary
Q16. This project engages all relevant expertise across disciplines, fields, and/or professions to produce research that advances translation.			**0.674**	Cross-Disciplinary
Q6. This project uses a multi-disciplinary approach.			**0.667**	Cross-Disciplinary
Q18. This project leverages knowledge integration to develop more holistic findings that are therefore more relevant to real-world applications.			**0.542**	Cross-Disciplinary

### Utility of Factors for Discriminating Between TS and TR

Prior to examining the utility of the factors for discriminating between TS and TR projects, the expert panel reviewed all 26 submitted projects and reached consensus on the project type for 12 projects (six TS and six TR; see Supplementary Materials for examples). These 12 projects were subsequently utilized to examine whether each of the factors was able to discriminate between TS and TR (see Table 3). As shown in Table 4, the *t*-tests were statistically significant for Generalizability/Efficiency and Disruptive Innovation, but not for Team Science.

**Table 4.** Utility of factors for distinguishing between translational science and translational research

Factor	No. of items	TS Projects *N* = 23 Mean (SD)	TR Projects *N* = 23 Mean (SD)	*t*	*p*
Generalizability/Efficiency	7	5.43 (2.12)	1.47 (1.72)	6.92	< 0.001
Disruptive Innovation	5	4.21 (1.08)	3.21 (1.41)	2.69	0.010
Team Science	4	1.08 (1.41)	1.82 (1.23)	−1.89	0.06

Factor scores were computed as the sum of items marked “agree.” *N* = 23 refers to the number of coded research proposals, which represents a function of the number of pilot proposals (6 TS and 6 TR) and the number of coders who evaluated each proposal (within each project type, five projects were evaluated by four coders and one project was evaluated by three coders).

**Figure 1.**
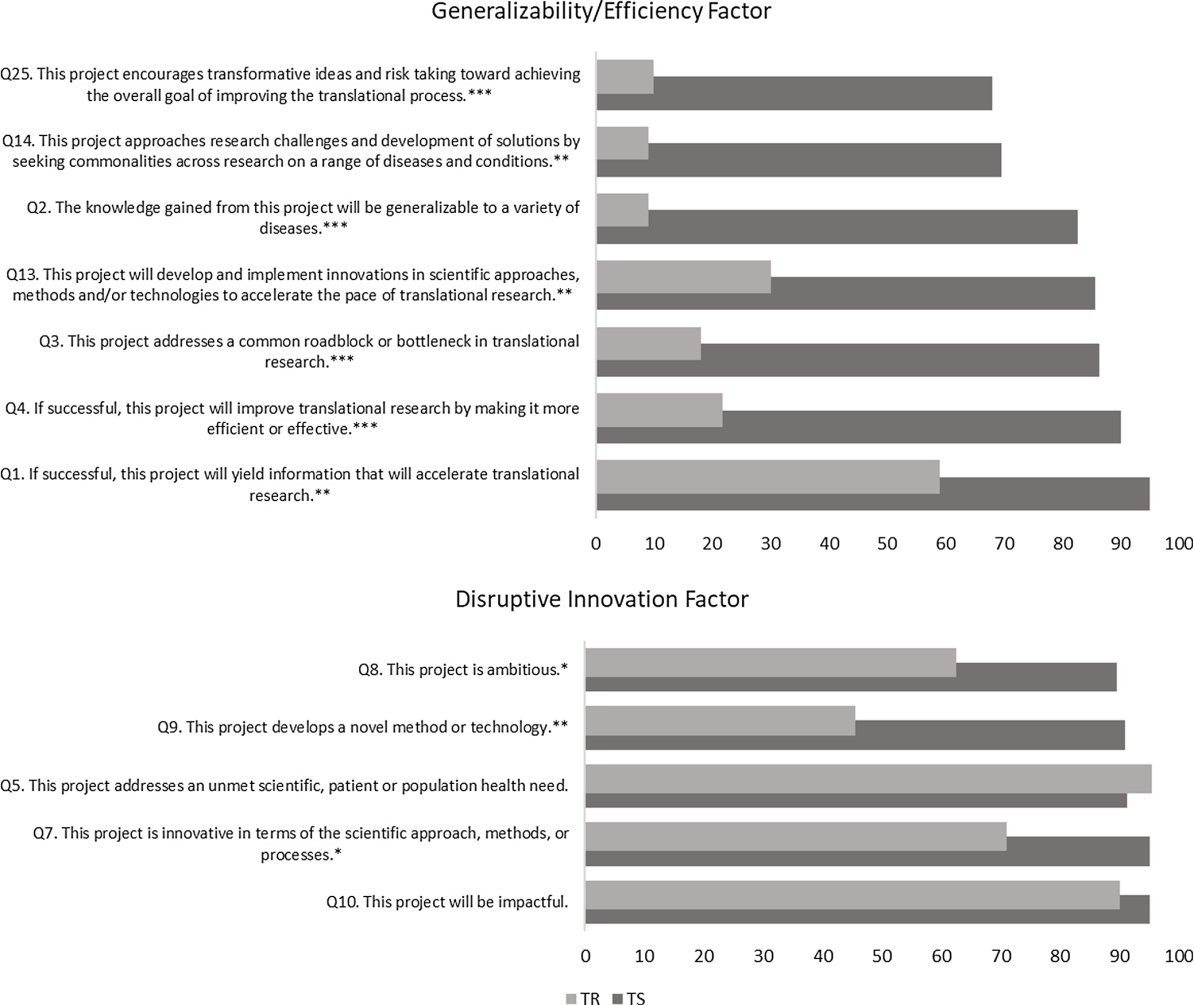
Percent agreement with questions (by factor) by project type. TS = translational science; TR = translational research. * *p* < .05; **p < .01; ***p < .001.

To aid in interpretation of the findings, the percent agreement with each question in the two factors that showed promise for distinguishing between TR and TS is illustrated in Figure 1. As might be expected given the large percent of the variance accounted for by the Generalizability/Efficiency factor, the large differences in percent agreement between TS and TR projects on each of the seven questions in this factor demonstrate that the principles of *Generalizable Solutions* and *Speed and Efficiency* have high utility for distinguishing TS from TR (Chi-Square analyses showed that all differences were statistically significant). Within the Disruptive Innovation factor, the percent agreement was significantly higher for the TS projects on the questions that assessed project ambition, development of a novel technology, and innovation. There was no difference in agreement with the questions that tapped into whether the project addressed an unmet need or would be impactful.

## Discussion

The purpose of this project was to explore whether a set of TS principles set forth by NCATS might be useful in distinguishing TS from TR. The results identified seven Translational Science Principles questions that showed evidence of considerable promise for aiding CTSA Pilot Program administrators in making this determination. These items map onto two of the key TS principles defined by NCATS: (1) produce cross-cutting solutions for common and persistent challenges (*Generalizable Solutions*); and (2) enhance the efficiency and speed of translational research (*Efficiency and Speed*). Based on the comparisons of percent agreement with these seven items across TS and TR projects, we suggest that these seven questions may be useful to Pilot Program administrators both in educating investigators about the nature of TS and in making the qualitative determination as to whether a particular project is aligned with TS principles. Future work building on these findings may generate a checklist that will make this determination more reliable across administrators and hubs.

It is noteworthy that in the time since the Translational Science Principles questions were developed, NCATS has revised the organizational framework of the TS principles posted to their website. The original version was also published in the Journal of Clinical and Translational Science in 2022 [3]. The two TS principles represented in the Generalizability/Efficiency factor fell, respectively, under the two subdivisions of principles that were referred to as “scientific” (including *Generalizable Solutions*) and “operational” (including *Efficiency and Speed*). As a whole, the scientific principles focused on features directly related to research question selection, research approaches, and rigorous methods while the operational principles focused on how team functioning, organizational environment, and the culture of science influence the research. The current version (as of December 2023) of the Translational Science Principles posted to the website [15] omits these higher-order categories of “scientific” and “organizational.” This evolution of the way that the principles are depicted reflects the dynamic and still unfolding understanding of how best to communicate and utilize these principles. Our study suggests that a further refinement might involve creating something of a hierarchy of principles to distinguish between those characteristics of the research that are necessary or defining features of TS (i.e., *Generalizable Solutions* and *Efficiency and Speed*) and those features that are equally likely to be found in TR projects (i.e., *Focus on Unmet Needs; Cross-disciplinary Team Science; Boundary-crossing Partnerships*).

We note that the principles of *Generalizable Solutions* and *Efficiency and Speed* are at the level of intended outcomes, whereas other principles set forth by NCATS address specific strategies expected to facilitate the achievement of these outcomes. For example, all the questions that loaded onto the Team Science factor in our study, the only factor that failed to distinguish between TS and TR at all, mapped onto the principle of *Cross-Disciplinary Science*. While there is evidence that research produced by cross-disciplinary teams has better outcomes, including greater productivity and scientific impact, compared with less distributed teams or individual scientists [25], cross-disciplinarity is not a necessary feature of TS. The invention, for example, of a more efficient cell-sorting technology may have dramatic implications for the efficiency of research across a wide range of diseases but may not involve collaboration across multiple disciplines. Our study suggests that whether or not a particular research project features cross-disciplinary science is not a useful distinction when determining whether or not that project should be designated as TS. That said, there may be other motivations at the programmatic level for considering cross-disciplinarity when making funding decisions. Thus, it is important to make a distinction between determining project eligibility in terms of whether it meets the definition of a TS project and project fundability in terms of whether it will be consistent with programmatic objectives.

Whereas our data show that all the questions in the Generalizability/Efficiency factor have utility for distinguishing between TS and TR and none of the questions in the Team Science factor do so, the findings were mixed for the Disruptive Innovation factor. Of the five questions in the Disruptive Innovation factor, three showed significantly higher percent agreement for TS as compared to TR projects: This project develops a novel method or technology; This project is ambitious; and This project is innovative in terms of the scientific approach, methods, or processes. These questions echo the statement by Christopher P. Austin, former NCATS Director, that TS studies “must develop a technology or insight or paradigm to improve the efficiency or effectiveness of a rate-limiting translational roadblock (p. 1634) [14].” The other two questions in the Disruptive Innovation factor relate to whether the project is impactful and whether it addresses an unmet clinical need. As noted above, these qualities may be found in TR projects which, though not focused on the science of translation, may nevertheless be ambitious in their aims and address an unmet clinical need.

We must emphasize here that the results of this study should not be used to discourage research efforts or projects at specific points along the continuum of research translation. As many have advocated [1,11,26], TS principles can be applied across the translational spectrum, including research that seeks to translate findings from clinical trials into everyday clinical practice, research translating new findings into community practice, and translation of new scientific knowledge into disease prevention population or global health strategies. It has further been argued that TS is not unidirectional; instead, it can be applied to both bench-to-bedside and bedside-to-bench research [14]. Such bedside-to-bench translational research efforts have led to many biomedical breakthroughs over the past 100 years [1,26]. There is nothing in the current study to suggest that where a study falls along the translational spectrum should determine whether the research meets the definition of TS.

A 2008 publication reviewing the history and future trends of what the author called translational science but in fact conflated with translational research stated that “The formal identification of translational science can be expected over the next 10 years or so..(p. vii) [4].” This prediction has generally come true, but TS is still in its nascent stage of development [1,3]. There are still many misconceptions about the distinctions between TR and TS [16,17]. Multiple terms and meanings of TR exist in biomedical research [13] and researchers in different scientific domains have different perspectives and practices [12]. To advance the definition of TS currently endorsed by NCATS [3,27], the Pilot Program administrators within CEREC have here endeavored to identify which of the TS principles are central to the TS/TR distinction. Pilot Program administrators are in a unique position to disseminate these principles as they issue their new calls for proposals under the new FOA. We anticipate that the seven items in the Generalizability/Efficiency factor may prove to be useful not only for selecting applications that satisfy the mandate to fund TS projects but also for educating the investigator community about the differences between TS and TR. As institutional culture often is the product of a research institute’s infrastructure, policy, norms, and leadership [28], administrative leaders, including CTSA Pilot Program directors and managers, can play a critical role in advancing TS by fostering a broader understanding of what it is and what it is not within the academic research community.

This study has some limitations that should be considered in generalizing the findings. One limitation of the findings presented here is that the 26 CTSA-funded pilot studies that informed the factor analysis were projects that were funded before the release of the recent NIH FOA. Accordingly, pilot study proposals included in this study were expressly not written to conform to the definition of TS that has since become more coherent and more widely understood. In their reviews of the projects, members of the expert panel in this study noted that a number of the studies on which they could not reach consensus (i.e., was it TS or TR?) could have been framed in such a way that the TS nature of the project was made explicit. As written, however, the implications of the research for future research efficiency and/or the generalizability of the research across multiple diseases were left unstated. Future cohorts of CTSA pilot applications will no doubt be more likely to include language that highlights the TS elements of the proposed work, which may change the relative prominence of each TS principle in terms of its ability to distinguish between TS and TR. A second limitation is that a “gold standard” of what defines a TS project does not exist, so we relied on expert consensus to identify TS vs. TR projects. Our definition of a consensus allowed for a single dissenter on the expert panel, so there is still room for debate as to the classification of a few of the studies. Nevertheless, even with this potential for uncontrolled variance in our analyses, we identified a cluster of seven questions that show strong promise for distinguishing between the two project types. A third limitation relates to the still-evolving delineation of TS principles as promoted by NCATS. In the December 2023 version, there is a principle that was omitted from earlier versions and therefore, omitted from our study: *Prioritize Diversity, Equity, Inclusion, and Accessibility*. We posit that this principle is equally relevant to both TS and TR and would be unlikely to aid in the distinction between the two, but this is a hypothesis that has yet to be empirically tested. An important additional caveat to take into consideration is the arbitrary five-page limit that we placed on the research materials that were submitted for scoring. This limit was determined for pragmatic considerations of the time burden placed on coders, but the result was that materials normally included with proposals (e.g., investigator biosketches, letters of support) were omitted. It is entirely possible that these supplementary materials may have provided additional information that would have enabled coders to form an opinion on items that were checked “insufficient information to determine” in the present study. Future work building on these findings should consider including all proposal materials to inform the coding process.

In conclusion, we leveraged the collective experience of 12 CTSA Pilot programs to identify a set of seven questions mapping onto two TS principles that hold promise for informing the determination of whether a proposed research project meets the definition of TS. These seven questions map onto the principles of *Generalizable Solutions* and *Efficiency and Speed*. CTSA Pilot Program administrators may find it helpful to use these items to inform their evaluation of proposals and to make the determination as to whether they are eligible for funding. Operationally, it may be useful to incorporate these items into application and/or review materials. While adherence to these principles may be necessary to advance a proposal for consideration, such adherence is unlikely to be sufficient to warrant funding. Individual hubs, NCATS, and NIH as a whole may aspire to fund projects that adhere to additional principles such as prioritizing diversity, equity, inclusion, and accessibility and/or leveraging cross-disciplinary science. Moreover, a number of the principles that are listed on the NCATS website as principles of TS are hallmarks of robust health science in general: prioritizing initiatives that address unmet needs and using bold and rigorous research approaches. Thus, all the principles set forth by NCATS may be of value for evaluating the fundability of proposed research projects. Our study has identified two of these principles that appear to be most useful in making the distinction between TS and TR.

## Supporting information

Schneider et al. supplementary material Schneider et al. supplementary material

## Supplementary material

The supplementary material for this article can be found at https://doi.org/10.1017/cts.2023.700.
