# Supplementary material for: Distinguishing between translational science and translational research in CTSA pilot studies: A collaborative project across 12 CTSA hubs
Source: J Clin Transl Sci. 2023 Dec 18;8(1):e4. doi: 10.1017/cts.2023.700 (PMC10877521; doi:10.1017/cts.2023.700)
Supplement: Schneider et al. supplementary material [file S2059866123007008sup001.docx]

| Supplementary Table 1. Participating CTSA Hubs | | | | | |
| --- | --- | --- | --- | --- | --- |
| Hub Institution | **First year of CTSA funding** | **No. CTSA Pilot^1^ Applications in 2021** | **No. CTSA Pilot^1^ Awards funded in 2021** | **Total CTSA Pilot^1^ funds awarded in 2021** | **CTSA Size per NIH Funding Opportunity Announcement (FOA)** |
| Harvard Catalyst | 2008 | 48 | 10 | 500,000 | Large |
| Medical College of Wisconsin^2^ | 2010 | 25 | 7 | 350,000 | Small |
| The Ohio State University^2^ | 2008 | 53 | 12 | 434,573 | Medium |
| University of Alabama, Birmingham | 2015 | 18 | 6 | 180,000 | Large |
| University of Arkansas for Medical Sciences^2^ | 2010 | 15 | 6 | 270,065 | Small |
| University of California, Irvine^2^ | 2010 | 18 | 8 | 200,000 | Small |
| University of Washington^2^ | 2007 | 37 | 8 | 315,000 | Large |
| Virginia Commonwealth University^2^ | 2010 | 8 | 4 | 100,000 | Small |
| Children’s National Hospital and George Washington University^2^ | 2012 | 20 | 9 | 400,000 | Small |
| Tufts University^2^ | 2008 | 19 | 8 | 324,129 | Large |
| Duke University^2^ | 2006 | 33 | 2 | 243,595 | Large |
| Indiana University School of Medicine^2^ | 2008 | 24 | 9 | 674,470 | Medium |
| ^1^CTSA Pilots are projects that are at least partially supported with NCATS resources (direct, voluntary committed cost sharing, or both).  ^2^Contributed Pilot Study proposals for analysis in this study | | | | | |
